# Memory for Music (M4M) protocol for an international randomised controlled trial: effects of individual intensive musical training based on singing in non-musicians with Alzheimer’s disease

**DOI:** 10.1136/bmjopen-2024-095136

**Published:** 2025-10-20

**Authors:** Marcela Lichtensztejn, Anja-Xiaoxing Cui, Monika Geretsegger, Astri J Lundervold, Stefan Koelsch, Daniela M Pfabigan, Jörg Assmus, Elias Langeland, Marianna Ruiz, Carolina Tabernig, Ragnhild Eide Skogseth, Christian Gold

**Affiliations:** 1 Universidad de Ciencias Empresariales y Sociales, Buenos Aires, Argentina; 2 Department of Musicology, University of Vienna, Wien, Austria; 3 NORCE Research AS, Bergen, Norway; 4 Department of Biological and Medical Psychology, University of Bergen, Bergen, Norway; 5 Department of Endocrinology, Obesity and Nutrition, Vestfold Hospital Trust, Tonsberg, Norway; 6 National University of Entre Rios, Faculty of Engineering, Oro Verde, Entre Rios, Argentina; 7 Department of Geriatric Medicine, Haraldsplass Deaconess Hospital AS Surgical Clinic, Bergen, Norway; 8 Department of Clinical Sciences, University of Bergen Department of Clinical Medicine 1, Bergen, Hordaland, Norway; 9 Department of Clinical and Health Psychology, University of Vienna, Wien, Austria

**Keywords:** Dementia, Psychosocial Intervention, Electroencephalography

## Abstract

**Introduction:**

The number of people living with dementia is increasing worldwide. Alzheimer’s disease (AD) is the most common type of dementia. It typically manifests itself initially with cognitive impairment in the memory domain and gradually progresses towards affecting all activities of daily living. Active music interventions, particularly singing, may improve mood, social behaviour and quality of life. However, little is known about their effects on cognition, although some studies have provided promising results. The Memory for Music (M4M) project aims to fill this gap in research by measuring the effects of learning new songs on cognitive functioning. Specifically, M4M will examine memory for new songs in non-musician adults with AD after undergoing intensive versus minimal individual musical training based on singing novel songs.

**Methods and analysis:**

Home-dwelling adults with AD, 65 years or older, will receive 5 months of intensive intervention (2×/week) and 5 months of minimal intervention (1×/month). In a crossover design, participants will be randomised to receive either the intensive or minimal intervention first, with 2 months between the intervention periods. Participants will receive individual music lessons to learn new songs, provided by a music instructor with adequate training. The main outcomes will be measured at the beginning and end of each intervention period. General cognition will be measured with the AD Assessment Scale – Cognitive by an assessor blinded to the randomisation. Participants’ memory for music will be measured using the N400 component of electroencephalographic (EEG) event-related potentials in response to music stimuli. Additional outcomes evaluated during intervention sessions include mood and musical performance observations. With 113 participants randomised, the trial will have 80% power to detect clinically meaningful effects. Relations between mood, memory for music and cognitive abilities will be examined, with sex, age, AD stage, previous musical training and education as covariates. M4M will be conducted in close collaboration between academic researchers, service providers and service users to ensure relevance and applicability.

**Ethics and dissemination:**

Dissemination of findings will apply to local, national and international levels. The study has been approved by the Regional Committees for Medical and Health Research Ethics in Norway (reference number 759936) and by Mautalén Salud e Investigación, CECOM in Argentina (register code 14412).

**Trial registration number:**

Clinicaltrials.gov, NCT06611878.

**STRENGTHS AND LIMITATIONS OF THIS STUDY**

- Our study moves beyond reminiscence-based intervention for memory stimulation since the participants will be actively involved in learning novel songs during singing-based musical training sessions.
- The study focuses on home-dwelling older adults with dementia, with the intervention delivered at participants’ homes. By using electroencephalogram technology that is portable, inexpensive, non-invasive, less demanding for participants than other brain imaging examinations, performed in a naturalistic setting, this study will reach people who are less mobile or live in remote areas, thus improving generalisability.
- As a multinational trial conducted in urban and rural settings in high-income and middle-income countries, results will be relevant across diverse societies.
- Due to the nature of the intervention, participants cannot be blinded.

## Introduction

 There are 55 million people living with dementia worldwide, and this number is expected to increase to 78 million by 2030 and 139 million by 2050.^1^ As the most common form of dementia, Alzheimer’s disease (AD) contributes to 60–80% of cases.^2^ AD is a neurodegenerative disease characterised primarily by progressive cognitive deterioration, with closely interrelated changes in emotional domains. The initial cognitive profile typically includes deficits in episodic memory, naming, semantic memory and verbal fluency.^3^ As the disease progresses, these fundamental cognitive impairments underlie and exacerbate broader symptoms, including disorientation in time and space, sleep patterns and mood, significant personality changes, communication difficulties, and motor disorders. This progressive cognitive decline eventually leads to loss of independence, with an increasing need for assistance in activities of daily living.^2^

Although cognitive symptoms typically appear years after the pathology is established in the brain,^4^ they tend to be the crucial trigger for seeking clinical attention. While these cognitive impairments represent the clinical manifestation of the disease, at the neuropathological level AD is characterised by extracellular amyloid beta plaques and intracellular neurofibrillary tangles of hyperphosphorylated τ protein. These proteins disrupt the communication between neurons, leading to neuronal loss and altered rhythmic patterns.^5^ Early cognitive impairment in AD has been linked to changes in the hippocampus, which relates to the impairment to encode new information and to retrieve it later. These biochemical changes in the hippocampus later spread to other temporal regions.^5^

Identifying and providing suitable interventions is essential to improve the quality of life of those with AD and may delay the progression of the disease. Music-making has shown promise in protecting cognitive function in older adults, suggesting reductions in brain ageing. Musical training has been consistently linked to both structural and functional improvements across multiple brain networks^6^,^8^ and brain plasticity induced by musical training has been documented across the lifespan.^9^ Active participation in music is thought to enhance the functional demands placed on auditory, visual, motor, memory, attention, emotional and reward circuits by promoting multisensory integration, cognitive engagement, learning and reward processing, thereby contributing to overall increases in brain activity in adults.^9^ It has even been suggested that music training might attenuate the decline of non-musical skills and reduce the risk of dementia, due to brain plasticity still present in ageing.^10^ Therefore, initiating musical training later in life may still yield cognitive benefits.^10^ Additionally, music interventions,^11^ particularly those involving singing,^12^ have shown a positive impact on mood, behaviour and quality of life and contributed to combating age-related cognitive decline^13^ in those living with dementia. People with AD, whether they are musicians or not, tend to have a well-preserved musical semantic memory for songs learnt prior to the onset of the disease, which may contrast sharply with their general cognitive functioning deficits.^14^,^17^ Musical semantic memory refers to ‘known’ melodies, that is, purely musical information stored and accessed independently (independent evocation evidenced by singing) or by sense of familiarity (SoF when listening to a melodic progression (recognition of the melody of a piece of music), regardless of the timbre or tonality, stripped of any non-musical contextual information (such as title, name of the composer, musical era to which it belongs, past event in which this melody was heard, etc). Therefore, this conception for musical memory involves the retention of musical information without the associated non-musical details.^18^

However, individuals with AD exhibit lower performance when becoming familiar with new music or when learning new songs compared with healthy non-musician adults. Healthy non-musician adults have been shown to recognise new musical material 24 hours later after being exposed three times to a list of 24 unknown musical extracts under three different conditions (with 1 min pause between each time): after the intake of a dopaminergic antagonist, a dopaminergic precursor and a placebo.^19^ Non-musician adults with AD in a moderate to severe stage are shown to need more than three repetitions for new items to display explicit encoding abilities. When repeatedly exposed to listen to instrumental music, they may need four sessions per week for 2 weeks to develop a SoF,^20^ or they may become familiar with a novel 10-line song in 8 weeks by being exposed to learn it during once a week singing workshops.^21^ However, there has been a case of a non-musician adult with moderate to advanced AD who displayed an outstanding and increasingly improved ability to learn new songs over 4 years by attending individual adapted music lessons based on singing. This improvement occurred despite an overall deterioration due to the progression of the disease.^22^ If the ability of people with AD to learn novel songs suggested in this case study is confirmed in a randomised controlled trial (RCT), it would pave the way for designing music training interventions in new ways that move beyond the well-studied use of familiar songs from their youth in a reminiscence-oriented approach.

Altered brain structure and function is well documented in patients with AD, and several markers have been suggested. Among others, event-related potentials (ERPs) assessed via electroencephalogram (EEG) recordings can be used to study aspects of how the brain processes information in people with AD.^5^ It has been consistently reported that adults with AD show impairments in the N400 component during tasks assessing lexical and linguistic semantic processing.^5^ The N400 component is a negative ERP deflection peaking between 250–550 ms after stimulus onset, with maximal distribution over the centroparietal electrode sites. It is commonly observed when semantic expectations are violated and presumably originates from posterior middle temporal and parahippocampal gyri.^5 23 24^

Its amplitude has been shown to be reduced and delayed in healthy elderly adults, with even smaller amplitude or prolonged latencies and altered topography in response to linguistic stimuli^25^ in those with AD compared with healthy controls.^5^ This likely reflects dysfunction of semantic memory processes.

An N400-like component (we will refer to it as ‘N400’ for simplicity) can also be elicited by non-verbal stimuli, for example by violations of expected notes in a melody. Several studies have used the N400 component to investigate the processing of meaning with music. In addition, one study has investigated musical memory using the N400, suggesting that the N400 may also serve as a marker of musical memory, although its timing and origin are less solidly established than in response to verbal language stimuli.^26^,^28^ Previous studies have compared the shared neural components in the processing of semantic meaning in language and music by studying the double dissociation between memory-based and rule-based knowledge.^27 29^ Using familiar and unfamiliar melodies modified with unexpected in-key or out-of-key notes, researchers manipulated memory-based and rule-based knowledge, respectively. A double dissociation occurs where memory violations elicit N400 components, while rule violations elicit early negativities with a shorter latency, suggesting an extension of the rule/memory dissociation in language to music.^27^ Although research shows preserved musical semantic memory in adults with AD, it remains unknown whether unexpected memory violations in familiar musical stimuli would elicit such an N400 component response in this population.

Summarising, there is compelling evidence of preserved musical semantic memory in patients with AD, suggesting that music-based interventions may contribute to delaying cognitive decline. Still, there is a lack of rigorous studies examining whether musical semantic memory for newly learnt songs can be developed in patients with AD and, if so, whether this ability is linked to general cognitive function. The overall aim of this clinical trial is to address this gap in research by exploring the connection between musical semantic memory and cognitive function and by this follow-up on results from a recent case study showing memory for newly learnt music despite clinical deterioration.^22^

Based on outcome measures from observations of mood and behaviour, cognitive test performance and EEG examinations, the present study aims to answer the following research questions:

1. Can an individual singing-based intervention improve AD patients’ musical semantic memory?
2. Does improvement in AD patients’ musical semantic memory relate to improvement of cognitive functioning?
3. Are improvements in musical semantic memory and cognitive functioning mediated by mood?

## Objectives

The primary objective of this study is to examine effects of intensive vs minimal individual musical training based on singing novel songs on memory for music and general cognitive functioning in non-musician adults with AD.

**Figure 1:**
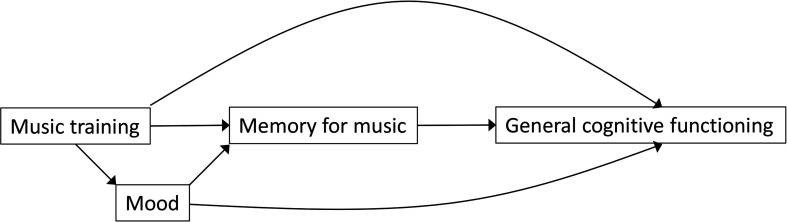
Path diagram of hypothesised intervention mechanisms and effects.

Secondary objectives are as follows:

- To determine the number of repetitions needed for independent recalling (total or partial) of new songs.
- To determine the quantity and quality of cues needed for such independent recalling.
- To determine EEG markers (eg, N400) related to the SoF for new songs.
- To examine if intensive individual training improves memory for music performance.
- To examine if effects on cognition and memory for music are mediated by mood and if effects on cognition are mediated by mood and memory for music (figure 1).

We hypothesise that participation in an intensive, individual singing-based intervention will improve the participants’ musical semantic memory, which in turn will be associated with improved cognitive function. This effect may be mediated by positive mood (figure 1).

Furthermore, if participants with AD show an N400 component in response to unexpected in-key violation of a newly learnt melody following the proposed intervention, it would indicate that musical semantic memory—as indexed through EEG measures—can be improved.

Findings gained from this study may have implications for evidence-based treatment planning and for designing opportune interventions aiming at reducing cognitive decline and improving the quality of life of people with AD. In line with the Global Action Plan on the Public Health Response to Dementia,^30^ the present study contributes by focusing on mechanisms of memory for music, particularly in an AD population and by investigating whether musical interventions have any impact on overall cognitive functioning. Additionally, the findings could be adapted and replicated in other parts of the world to design culturally sensitive approaches for this population.

## Methods

### Trial design and procedures

**Figure 2:**
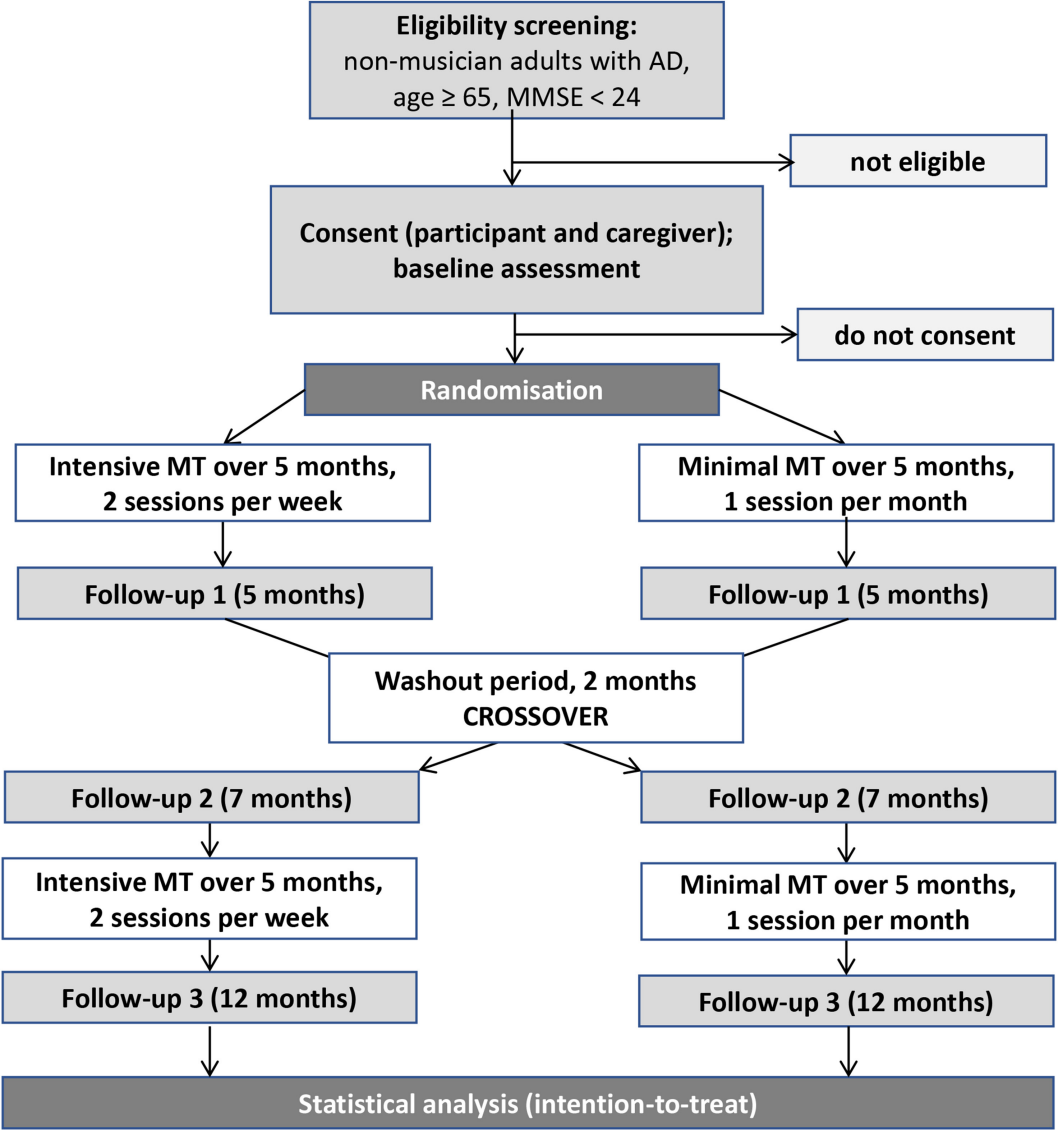
Consolidated Standards of Reporting Trials flow diagram depicting the design. AD, Alzheimer’s disease; MMSE, Mini-Mental State Examination; MT, musical training.

Memory for Music (M4M) is a prospective, experimental, crossover, before-and-after, international, assessor-blinded RCT (figure 2). A crossover design allows for comparison within subjects, accounting for effects of covariates and interindividual variation. Compared with a parallel design, a crossover design has the important advantage of obtaining the same statistical power with a smaller sample size. While crossover trials are less common in psychosocial interventions, this design is well suited to our aim: to compare different levels of intensity—minimal versus intensive—of the same musical training approach. The trial does not compare two distinct interventions or test the effect of intervention order but rather evaluates whether increased time and engagement with musical material enhances outcomes. To address potential carry-over, period or sequencing effects,^31^ we have taken the following precautions: (1) a wash-out period of 2 months is included between intervention phases; (2) different musical pieces are used in each phase, ensuring that any observed effects are attributable to training intensity rather than content familiarity and (3) the sequence of interventions (ie, order of interventions) can be included as a covariate in the statistical analysis to account for possible sequence effects.

The interventions are based on previously described and tested models used with musician and non-musician adults with AD.^20^,^2232 33^ In this context, the proposed approach builds on emerging knowledge and consensus related to the favourable outcomes secondary to the use of live music with this population. Eligible trial participants who have given consent to participate will be randomly allocated to receive musical training, either in a sequence of intensive followed by minimal intervention or vice versa with a washout period of 2 months in between the two phases. The intensive phase includes two sessions per week, minimal phase one session per month (figures2  3).

**Figure 3:**
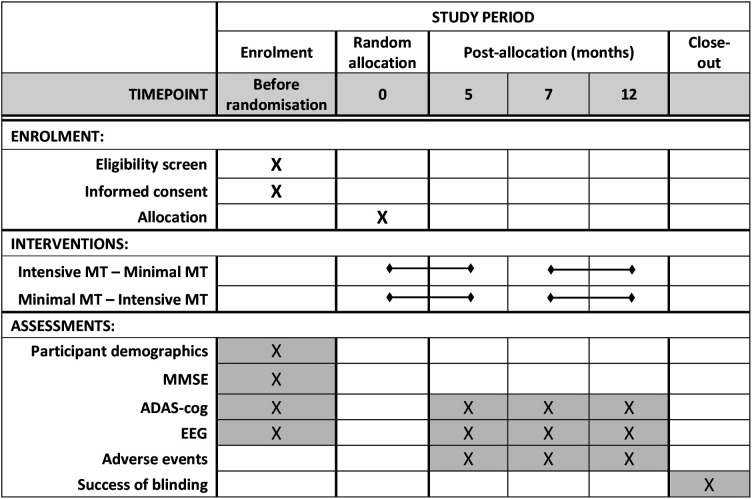
Standard Protocol Items: Recommendations for Interventional Trials diagram depicting the data collection schedule. ADAS-cog, Alzheimer’s Disease Assessment Scale – Cognitive Subscale; EEG, electroencephalogram; MMSE, Mini-Mental State Examination; MT, musical training.

Randomisation will be conducted centrally at NORCE, concealed from site investigators, using block randomisation with randomly varying block size, with separate lists for each site. Once a decision on inclusion has been made, informed consent obtained and baseline data collected, the randomisation result will be revealed to the instructors through an online system. Participants and instructors cannot be blinded due to the nature of the intervention. External evaluators will remain blinded after randomisation; success of blinding will be verified at the end of each participant’s participation. Treatment fidelity (adherence and competence) will be evaluated by external raters using video recordings. This study protocol is designed and reported in accordance with the Standard Protocol Items: Recommendations for Interventional Trials 2013 statement. Ethical approval has been obtained for Norway (Regional Committee for Medical Research Ethics South East Norway, 09 September 2024, reference number: 759936) and Argentina (Mautalén Salud e Investigación, CECOM, 28 February 2025, register code 14412). As funding for Austria was obtained after the initial submission of this protocol, approval for Austria has not been sought or obtained as of July 2025. Approval for this and any other future sites will be obtained from the responsible local ethics committees prior to starting recruitment there. The trial is registered in Clinicaltrials.gov, NCT06611878. Recruitment started in December 2024; the first participant was randomised on 1 April 2025. Recruitment is estimated to continue until autumn 2026, leading to October 2027 as the anticipated date for the last outcome assessment.

### Participants

We aim to recruit 113 non-musician adults with a diagnosis of probable or definite AD. To achieve the target sample size, it is necessary to recruit participants from multiple sites. Sites are located in Europe and South America (Bergen, Oslo, Kinn, Norway; Vienna, Austria; Buenos Aires, Argentina; see trial registration for updated list).

#### Eligibility criteria and enrolment

Participants eligible for the trial will be of any gender and ethnicity/nationality; aged ≥65 years; non-musician; have a documented diagnosis of probable or definite AD; and be home-dwelling in the vicinity of a study site. The research team will conduct the screening of the participants based on available results from neurological and neuropsychological examinations that support a profile compatible with AD, clinical history and musical engagement/history profile. Individuals with a confirmed non-AD dementia type (eg, vascular, frontotemporal, Lewy body, mixed or pseudodementia), suffering from other known neurological disorders (eg, Parkinson’s disease, multiple sclerosis, stroke), with known severe mental illness (eg, current major depression, bipolar disorder, major anxiety, schizophrenia) or with known severe hearing loss that is not compensated by hearing aids will be excluded. No cut-off score will be applied (table 1). For the purposes of this study, we consider as non-musicians those who have no history as professional musicians (see table 1 for history of revisions of this criterion). Individuals with a history as professional musicians will be excluded. Participants should live within a reasonable distance of a study site (eg, within ≈1-hour driving distance), understand the language(s) used at the study site and expect to be available for 1 year from enrolment. Those living in care home facilities will be excluded. They may be under pharmacological treatment for AD and other diseases; such treatment should be stable at least 8 weeks prior to inclusion and will be recorded. Study participation will be based on informed consent from the participant (the participant informed consent form as approved for Norway in November 2024 is available as online supplemental material). Enrolment will be voluntary and in response to a public recruitment call via media and advertisement flyers in day activity centres, hospital memory clinics and assisted living organisations.

**Table 1:** History of protocol amendments

Date	Amendment number	Version name	Amendment	Rationale
2022	–	Version 0 (original proposed by ML)	–	–
February 2023	–	Version 1 (original submitted to RCN; accepted for funding by RCN in 2023; and submitted to REK for ethics approval in 2024)	Design changed from parallel to crossover; (2) primary outcome changed to general cognition; (3) ERP stimuli specified further.	Improved power; (2) direct patient relevance in line with funding call; (3) improved clarity.
June 2024	1	Version 2 (this version; submitted for publication)	No MMSE cut-off.	MMSE scores depend on gender, age and education level. Clinical criteria are more meaningful.
June 2024	2	Version 2	Clarified definition of ‘non-musician’: removed ‘no intensive training in childhood or youth up to 20 years of age; ≤ 2 years of musical practice/training in adulthood’; retained only ‘no history as professional musician’.	Original additional criteria were unclear and did not work well across countries due to different education systems.
August 2024	3	Version 2	Recruit only participants with the ability to provide informed consent.	Original was questioned by Norwegian ethics board. Number without ability to consent expected to be very low among otherwise eligible participants.
August 2024	4	Version 2	Definition of regions of interest (ROIs) for ERPs: removed specific electrode names in favour of more general description (6 ROIs, 2×3 grid; exact time window and ROI to be adjusted).	Electrode names may not be applicable to different EEG systems used across sites.
August 2024	–	Version 2	ERP stimuli and other design aspects specified further (eg, only in-key violations); descriptions improved.	Further clarification of procedures.

EEG, electroencephalogram; ERP, event-related potential; MMSE, Mini-Mental State Examination; RCN, Research Council of Norway; REK, Regional Committees for Medical and Health Research Ethics.

#### Baseline data

Demographics data, performance on the Mini-Mental State Examination (MMSE)^34^ and baseline values of outcomes (described below) will be collected before random allocation. Participants’ history of musical training and general education will be recorded. The Music Engagement Questionnaire^35^ will be completed by participants and/or caregivers, to provide insight into participants’ everyday life engagement with music, which may relate to musical semantic memory.^36^ In addition, we will collect information about the language(s) spoken by the participants, the age at which each was acquired and the proficiency levels they had attained before starting to suffer from cognitive decline. Where necessary, this information will be collected with the help of their next of kin. Several outcomes will also be collected at baseline (see the Outcomes section and figure 3).

#### Interventions

Interventions will be conducted by an instructor with adequate qualification and experience in music teaching and dementia care. Instructors will ideally have a music therapy degree, but music educators with relevant experience and training, supervised by music therapists, may also be able to conduct singing interventions for dementia.^12^ Interventions will entail individual musical training based on singing to learn 10 randomly selected songs from a list of 20 novel songs specifically created for the purposes of this study. These songs will take into consideration adequate musical complexity, length of verses and chorus and high-frequency words for the lyrics. The structure of the complete lyrics of the song will have the following sequence (similar to popular music): first verse; second verse; chorus; repetition of the first two verses; chorus; repeat chorus; final cadence.

Participants will be randomly assigned to one of the two sequences: intensive intervention phase (two sessions per week) followed by minimal intervention phase (one session per month) or vice versa (figure 2**). They will receive individual musical training based on singing during both phases**, lasting 5 months each, with 2 months of no intervention in between. During both intervention phases, participants will learn and focus the singing practice on one novel song per month. That means a total of five novel songs will be offered in each phase. The order of the songs will be randomised across the 10 months of intervention.

The instructor will use live music by singing with the support of a harmonic instrument such as piano, keyboard, guitar or accordion to deliver the musical experiences. At all times, the instructor must provide appropriate and individualised support encouraging the participant to engage in the tasks without forcing him/her to do so. It is crucial that the instructor establishes an empathic relationship with the participant for the musical experience to be pleasant, rewarding, emotionally meaningful and conducive to learning. Each session will have a duration of 40–45 min and will be conducted according to a sequence of steps^22^ as follows:

#### Step 1: baseline mood observation

The instructor will show a set of icons (online supplemental Figure S1) to the participant to self-report his/her mood and will note the response on the Scoring Session Instructor Form (SSIF, online supplemental Form F1). If this is not possible, the instructor will complete it based on observed behavioural/gestural/verbal responses.

#### Step 2: warm up

The instructor will offer a 5 min warm-up which includes breathing exercises and vocalisations. Five breathing exercises will be performed at the beginning of the lessons to practise breathing, air dosing, as well as to promote relaxation and prepare the participant for singing. After this, vocalisations will be performed. They must be offered in a pitch range matched to the participant’s vocal range. It will entail a sequence of two vocalisations such as: (a) major thirds by ascending and descending steps, (b) perfect fifths by ascending and descending steps. The rhythmic pattern suggested is a dotted eighth note with a 16th note (online supplemental Figure S2), sung in a playful manner to promote positive mood.

#### Step 3: teaching the song

First, the instructor will introduce the new song by singing it through once. A key which is comfortable for the participant will be used in all cases. The instructor will then complete a 5-item Likert scale to describe the participant’s response (liking or disliking) to the new song based on observed behavioural/gestural/verbal responses (5-point Likert scale; Online supplemental Form F1). The instructor will then teach the song. By applying their own judgement, the instructor will decide which didactic resources and strategies are best to teach the song based on the participant’s preferred learning style and performance. The instructor might choose one of the following teaching strategies: (a) repeatedly singing the entire song together with the participant having the printed lyrics as support; (b) repeatedly singing together with the participant one line at a time until completion of the chorus first and then the verses of the song, having the printed lyrics as support; (c) without having printed lyrics, the participant will learn by imitation one line at a time or the entire song. If choosing the option of teaching one line at a time during the *intensive* intervention and one session is not enough to work on the entire song, the verses and the chorus will be learnt in two different consecutive sessions. Each line should be repeated between four and six times to provide opportunities to consolidate learning. If choosing the option of teaching one line at a time during *minimal* intervention, the instructor will begin teaching the chorus first, and if there is sufficient remaining time, will teach the verses. The instructor will offer the type and number of cues that the participant needs to successfully perform the task of learning and recalling the song. The instructor will use their own judgement to gradually decrease or increase cues and support depending on participant needs to successfully complete the singing task.

#### Step 4: singing the entire chorus

The participant will be invited to sing the entire chorus. The instructor will provide the type and amount of assistance/support as needed by the participant to successfully perform the singing task. This step should be repeated eight times.

#### Step 5: singing chorus in context

The participant will be invited to perform the entire song together with the instructor. The instructor will sing the verses solo, and the participant will sing the chorus solo. The instructor will provide support of the printed lyrics of the entire song. The participant may be inclined to join singing during the verses as well; the instructor will give space for him/her to do so. A brief musical instrumental introduction will be offered to establish the musical structure and character of the song and to orient the participant in tonality, metre, character and tempo of the song. The instructor will provide the type and amount of assistance/support as needed by the participant to successfully perform the singing task. This step should be repeated at least twice. At each song ending, instructor and participant may engage in a brief verbal exchange about the singing experience or the content of the song, which will work as a brief pause, release and distractor before repeating the full song a second time.

#### Step 6: practising previously learned new song

Starting in month two of the first musical training phase, the instructor will invite the participant to rehearse a novel song learnt in a previous month (table 2). The intervention will be conducted as described in *Step 5*.

**Table 2:** Combination of songs for rehearsal (example with minimal intervention first)

(a) Minimal intervention phase						
Task	Month 1	Month 2	Month 3	Month 4	Month 5	
New song	Song 1	Song 2	Song 3	Song 4	Song 5	
Rehearse	–	Song 1	Song 2	Song 3	Song 4	
**(b) Intensive intervention phase**						
Week	Task	Month 8	Month 9	Month 10	Month 11	Month 12
1	New song	Song 6	Song 7	Song 8	Song 9	Song 10
	Rehearse	–	Song 6	Song 7	Song 8	Song 9
2	New song	Song 6	Song 7	Song 8	Song 9	Song 10
	Rehearse	–	–	Song 6	Song 6 or 7	Song 6, 7 or 8
3	New song	Song 6	Song 7	Song 8	Song 9	Song 10
	Rehearse	–	Song 6	Song 7	Song 8	Song 9
4	New song	Song 6	Song 7	Song 8	Song 9	Song 10
	Rehearse	–	–	Song 6	Song 6 or 7	Song 6, 7 or 8

This is an example with minimal intervention first. The order is reversed for those randomised to intensive first.

#### Step 7: session closure

The purpose is to relax and bring the session to closure. If the participant is available and willing to do so, he/she will choose a familiar favourite song and will sing it once together with the instructor. The instructor will provide support of printed lyrics if needed. The instructor will accompany the participant with the harmonic instrument (piano, keyboard, guitar or accordion) for the participant to accomplish this task. A comfortable tonality for the participant will be used in all cases. The instructor will decide moments of solo and tutti depending on the participant and will provide a musical context by sensitively responding to the musical and communicative expressions of the participant.

#### Step 8: final mood observation

The instructor will again use a set of icons (online supplemental Figure S1) and note the response or observed behaviour (Supplementary Form F1) to describe the participant’s final mood as in *Step 1*.

#### Step 9: session rating

After the session, the instructor will complete the remainder of the SSIF (online supplemental Form F1) to record what happened in the session, including the participant’s responses and performance, title of the intervention song, title of favourite song, duration of the session, the degree of independent evocation and SoF.^20 37^ The participant’s independent recall will be rated as follows: (a) needs full support; (b) needs intermittent support to complete each line; (c) needs support in one syllable/word per line; (d) fully independent. To record independent evocation, the instructor will indicate with an ‘X’ when the first independent recall occurs during the session, which means they will mark only one cell in the form depending on type of recall and in which repetition it occurred. If the degree of independent recall changes over the same session, the instructor will also indicate with an ‘X’ the maximum degree of independent recall where it applies. Otherwise, if remaining stable, ‘no change’ will be recorded on the SSIF.

Ratings of mood and SoF will also be analysed as outcomes (see the Behavioural outcomes section).

#### Assessment of intervention fidelity

External evaluators blinded to intervention type and session number will rate selected sessions at specific points of the training (last session of each month and when the instructor’s SoF rating changes). To that end, sessions will be video recorded to allow external blinded evaluators to audit the session to determine adherence to the intervention manual and competence by completing the Intervention Fidelity Form (online supplemental Form F2).

### Outcomes

The two primary outcomes, participants’ scores on the ADAS-cog^38^ and the N400 response measured using EEG, will be assessed before and after each musical training phase (figure 3). Behavioural assessments of participants’ familiarity with the song and song performance will be conducted by the instructors in every session and by external evaluators in selected sessions at specific points of the training (last session of each month and when the instructor’s SoF rating changes). Secondary outcome measures include the behavioural assessments, participants’ self-reported mood during each intervention session and any reported adverse events.

### Primary outcomes

#### Cognitive outcome: ADAS-cog

Before and after each musical training phase, a neuropsychological assessment battery (ADAScog)^38^ will be administered to all participants by a trained psychometrician blinded to the intervention allocation. The scale consists of items to assess the following domains: language; memory; praxis and orientation. The standard ADAS-cog includes the following 11 subtests, with a total scoring range from 0 (no impairment) to 70 (most severe impairments): word recall (0–10); commands (0–5); naming (0–5); constructional praxis (0–5); ideational praxis (0–5); orientation (0–8); word recognition (0–12); remembering word recognition test instructions (0–5); spoken language ability (0–5); comprehension of spoken language (0–5); word-finding difficulty (0–5). The ADAS-cog score is based on the number of errors made within each subtest. Two versions will be included: one administered at baseline and at the 7-month assessment; another at the 5-month and 12-month assessments. In a retest situation, the minimum clinically important difference (MCID) is 3.^39^ The ADAS-cog takes 30–35 min to administer. The total score of the ADAS-cog at the end of each intervention will be used as the *primary clinical outcome measure*, and the scores on the 11 sub-domains as secondary clinical outcomes. We aim to administer the ADAS-cog within 7 days before the first and after the last musical training session of each intervention phase.

#### Neurophysiological outcome: N400 amplitudes

**Figure 4:**
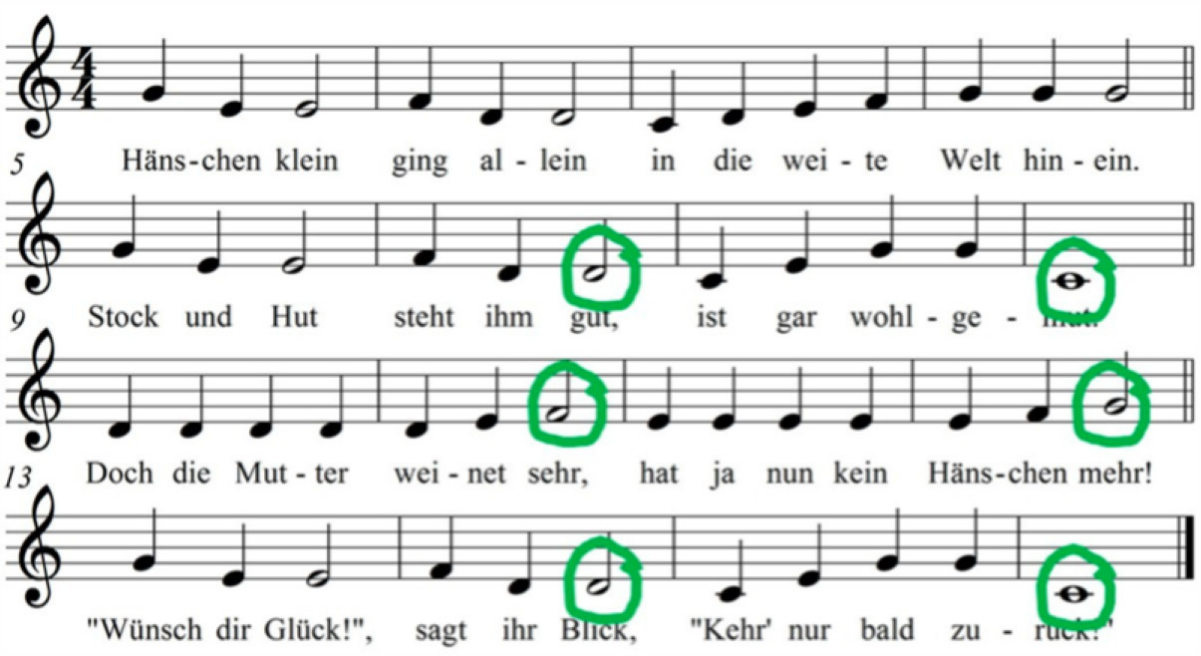
Example of a simple melody with possible places for in-key violations to detect N400 responses. Of note, actual stimuli will be novel songs specifically composed for this study, which will initially be unknown to all participants. This example of a well-known children’s song is shown for illustration purposes.

At the beginning and end of each intervention phase (figure 3), brain and behavioural response (SoF) during music recognition tasks will be recorded and analysed as outcome measures. We seek to determine the N400 component related to the in-key violations (see example in figure 4) in songs learnt during the intervention period and the potential correspondence with the behavioural observations. If participants do develop musical semantic memory through our intervention, we expect them to also show an N400-like component to in-key violations in familiarised melodies, that is, those melodies that are introduced and learnt during our intervention, but only after the intervention. If an N400 component is detectable during these in-key violations of a newly learnt song, it will indicate memory of the new song even if the participant is unable to adhere to a behavioural task or display an observable SoF.

##### EEG protocol

Each site will collect EEG with at least 32 channels based on the 10–20 system. Preferably, active EEG systems that are less sensitive to interference from other sources will be used, which is especially important when recordings are made outside shielded rooms (eg, at participants’ homes). EEG recordings will start with a resting state measurement to familiarise participants with the situation of the EEG data collection. For the purposes of our study, a previously used paradigm^27^ is adapted to have in-key violations in familiarised/to-be-familiarised and unfamiliarised melodies only to assess reactions to memory-based knowledge. No out-of-key violations will be introduced. The in-key violations will be introduced at multiple places throughout each melody, which will consist of at least 32 bars. No violations will be introduced in the first four bars, to allow participants sufficient time to recognise the melody. A total of 10 melodies will be played for each participant, of which half are learnt in an intervention period (familiarised/to be familiarised). In each of the melodies, we will include at least eight in-key violations. ERPs in response to these in-key violations will be compared with ERPs in response to unchanged notes in structurally similar places in each melody. After each melody, applause will sound. Participants will be instructed to indicate via a button when they hear the applause. We estimate that the paradigm will last about 20 min. Half of the melodies will be and remain novel or unfamiliar, that is, they will not be introduced during the intervention. The other half of the melodies will be novel at the first EEG assessment but be introduced during the intervention and thus be considered familiarised at the second EEG assessment (familiarised/to be familiarised). At least 40 in-key violations will appear in novel melodies, and at least 40 in-key violations will appear in familiarised melodies. EEG assessments will be conducted within 7 days before the first and after the last musical training session of each intervention phase. If memory for music is gained through the intervention, we expect more pronounced N400 amplitudes to in-key violations in familiar melodies but not to in-key violations in unfamiliar melodies after the intervention. Thus, we expect an interaction effect of time (before vs after intervention period) and melody type (familiarised/to be familiarised vs unfamiliarised) on N400 amplitude variation.

##### EEG data analysis

EEG raw data will be converted to MATLAB-readable files and preprocessed within a standard EEG evaluation software package (e.g. EEGLAB) according to recommended processing pipelines. General EEG preprocessing steps will include bandpass filtering, artefact rejection, including the removal of noisy channels and time segments, and eye movement components identified via independent component analysis,^40^ before data epochs will be extracted for trials with or without memory violations for familiarised and unfamiliarised melodies. We will examine differences in mean amplitude in response to in-key violations and unchanged notes at six regions of interest (ROIs) based on previous research in a two-by-three grid with two levels of anterior/posterior and three levels of laterality (left, mid and right).^27 29^ The exact time window and ROI studied may be adjusted given the potential for an altered latency and amplitude of the N400 component in the participants^25^ and the use of different EEG systems between sites using cluster-based permutation tests.

### Secondary outcomes

#### Behavioural outcomes: mood, SoF and singing performance

*Mood* (Step 1 and 9 in Interventions) at the beginning and end of each session will be measured on 5-point Likert scales.

SoF with the current song will be assessed using the SoF scale,^20 37^ a 6-point Likert scale. It will be completed by the instructor at the end of each session (online supplemental Form F1) and by the external evaluator after selected sessions (online supplemental Form F3). For this study, a score of 4 or above will be considered as the threshold to determine familiarity-based recognition.

*Performance* as observed by an external evaluator based on video recordings will be analysed in selected sessions using the Participant Performance Cue Indicator Form (online supplemental Form F3). It yields a score for chorus solo (range 8–32, where 32 is best) and for chorus in context (2–8, where eight is best).

#### Adverse events

Although AEs of music interventions are rare,^41^ AE assessment is an important part of the safety aspect in all RCTs. We will ask participants or relatives at each assessment time point about the occurrence and a description of any serious or non-serious AEs. Potentially study-related serious AEs, as well as unblinded frequency counts of any AEs, will be shared and discussed with the data monitoring and safety committee (DSMC) through the trial statistician.

### Sample size and power, data monitoring and statistical analysis

#### Sample size and power

Although cognitive outcomes have been of secondary interest in music interventions in dementia so far,^11^ there is some research to suggest a plausible effect size. Our systematic review found a mean d=0.29 (95% CI 0.02 to 0.57) of active music therapy on global cognition, based on 3 RCTs (total 209 participants); in contrast, no effects were found for music listening.^42^ A study of group-based singing found similar effects.^43^ Analyses in the Cochrane review did not distinguish between active and listening interventions.^12^ The ADAS-cog was only used in one included study,^12^ although it is more sensitive than the MMSE^44^ and has a defined MCID.^39^ With a conservative assumption of SD=10 (a large sample had SD=6.42),^44^ the MCID of 3 corresponds to an effect size of d=0.30, similar to our previous review.^42^ We further assume a within-subject correlation of r=0.5 between intervention periods; this reflects a conservative and plausible estimate of consistency across repeated cognitive assessments in this population and is consistent with recommended practice in crossover trial planning.^45^ Together with an assumed attrition rate ≤20%, and aiming for 80% power with a two-sided 5% significance level, this leads to a required number of 113 participants to randomise (90 after drop-out). Similar power would be reached for slightly smaller d and higher r or vice versa; a higher power is used if the SD is smaller. In contrast, a parallel trial would require 440 participants to achieve the same power for the same effect size. Recruitment and follow-up rates as well as data quality will be monitored and discussed with the DSMC to ensure target sample size and power.

#### Statistical analysis

Sociodemographic and diagnostic features will be analysed via descriptive methods (mean (SD), range, n (%)). Outcomes will be analysed on an intention-to-treat basis (analysing all participants as randomised, regardless of actual participation), which provides a conservative estimate of effects, and additionally on a per-protocol basis (analysing participants according to their actual participation in the trainings). We estimate that participants need to receive around 90% of the sessions during intensive musical training intervention for learning effects to occur. Multiple imputation will be used if attrition is higher than expected. Continuous outcomes will, following a graphical examination of normality, compare the post-test difference between interventions within each participant, adjusted by the difference in pre-tests within each participant in a hierarchical ANCOVA model with participant and site as random effects. The ANCOVA model ensures optimal use of baseline measures to improve precision and avoids bias from randomly occurring baseline imbalance.^46^ The model can be written as (*Y_Ti_ – Y_Ci_) = β_T_ + γ(X_Ti_ – X_Ci_*), where *Y* is the post-test, *X* is the pretest, *T* is the treatment, *C* is the control condition, *γ* represents the influence of the pretest and the intercept *β_T_* represents the treatment effect.^46^ However, as this model does not address possible carry-over effects of the initial treatment (see Trial design and procedures), we will also include the randomisation (intervention sequence) in the model. Note that the model requires complete data for a given outcome at all four time points. Imputation is not recommended for missing outcomes because the estimation of the model already makes optimal use of the existing data. Secondary analyses will include additional covariates/subgroups such as sex, age, AD stage, history of musical training, general education, music engagement and interventionist’s background training (music therapy vs music education). The role of mediators will be analysed with a series of regression models (as depicted by each arrow in figure 1). Data will be analysed using R (r-project.org). A detailed statistical analysis plan will be developed before the beginning of data analysis.

### Patient and public involvement

The focus and research questions of this study are closely aligned with public guidelines emphasising the need to improve the quality of life and care for home-dwelling people with dementia.^47^ The study design and implementation strategies have been developed in close cooperation with societal partners such as municipal healthcare providers, clinicians and music interventionists in Norway and Argentina who have advised on relevance and feasibility of outcome measures, recruitment plans and intervention components. Their input was crucial in efforts to keep the burden caused by assessment procedures and interventions manageable for participants and their family members. Stakeholder partners will be particularly important in disseminating findings to clinical audiences and service user organisations, ensuring that study findings can be effectively implemented. Study participants and their carers will be notified about study findings via newsletters in addition to continuous updates available to the public through the project website and social media channel.

## Discussion

As the most common form of dementia, AD is one of the major causes of disability and dependency among older people. A global action plan issued by the WHO aims to ‘improve the lives of people with dementia, their carers and families, while decreasing the impact of dementia on them as well as on communities and countries.’^30^ Based on previous evidence from trials on music and dementia with promising effects on mood and behaviour problems, M4M focuses on cognitive outcomes in home-dwelling older adults with AD, thus aiming to prevent or decelerate further decline in their abilities. Using an intensive singing intervention to increase the ability to learn and recall new songs, this trial is in line with the goal for people with AD to experience greater mastery and to live active and meaningful lives with cultural activities adapted to individual interests and needs.^30^ The intervention is relevant for all genders and can be tailored to different cultural backgrounds. Providing individualised services to home-dwelling service users is in line with the aim of enabling people with AD to live with home care outside of care facilities as long as possible.

M4M will be the first study to rigorously examine musical semantic memory for newly learnt songs in patients with AD receiving individual musical training. By combining behavioural and EEG measures and relating them to performance on neuropsychological tests, we will further be able to explore how musical semantic memory may benefit cognitive functioning. Through EEG measurements, changes in brain processing in people with AD will be examined as a potentially more sensitive marker than behavioural and verbal responses to predict clinical outcome (figure 1). Our results may thus help move the field towards finding patient-tailored therapies.^48^

Finding ways to alleviate dementia symptoms, understanding the mechanisms by which potential interventions work and exploring the participant-specific factors that may contribute to intervention success are all crucial to cushion the enormous socioeconomic impact that dementia has. We expect to establish memory for music as an important mechanism and predictive marker of clinical improvement. In addition, we will also contribute to our fields’ understanding of the neural bases of musical semantic memory as well as the structure of musical semantic memory. M4M features interdisciplinary collaborations between neuropsychologists, musicologists, music therapists, music educators, trial methodologists, bioengineers, neuroscientists and biostatisticians. It will contribute to future practice development in dementia care and to theory and research in the fields of music, health, ageing and neuroscience. Findings will be directly applicable to clinical practice and will have a valuable impact for individuals with AD, their family members and practitioners working with them.

## Supplementary material

10.1136/bmjopen-2024-095136 online supplemental file 1

10.1136/bmjopen-2024-095136 online supplemental file 2
