# Supplementary material for: Memory for Music (M4M) protocol for an international randomised controlled trial: effects of individual intensive musical training based on singing in non-musicians with Alzheimer’s disease
Source: BMJ Open. 2025 Oct 20;15(10):e095136. doi: 10.1136/bmjopen-2024-095136 (PMC12542587; doi:10.1136/bmjopen-2024-095136)
Supplement: online supplemental file 1 [file bmjopen-15-10-s001.docx]

**SUPPLEMENTARY MATERIAL – MEMORY FOR MUSIC PROTOCOL**

**Supplementary Fig. S1. Likert Scale: Mood**


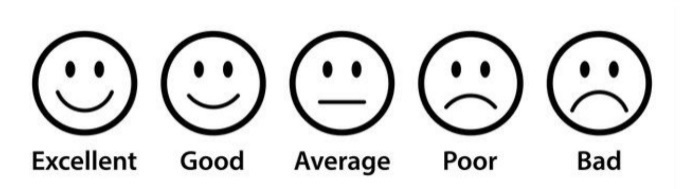


**Supplementary Fig. S2. Pattern for vocalizations**


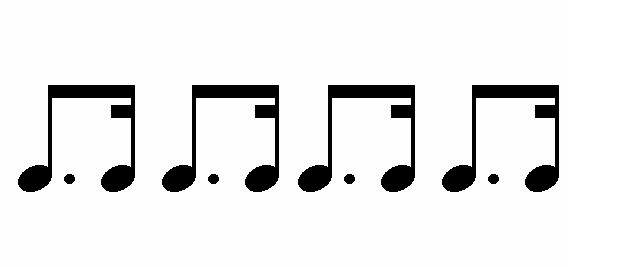


**Supplementary Form F1. Scoring Session – Instructor Form (SSIF)**

**Participant # ________________ Instructor: ____________**

**Date:** dd/mm/yyyy **Month #: ______ Lesson #: ______ Duration: _______** minutes

**Baseline mood** (check which one applies)


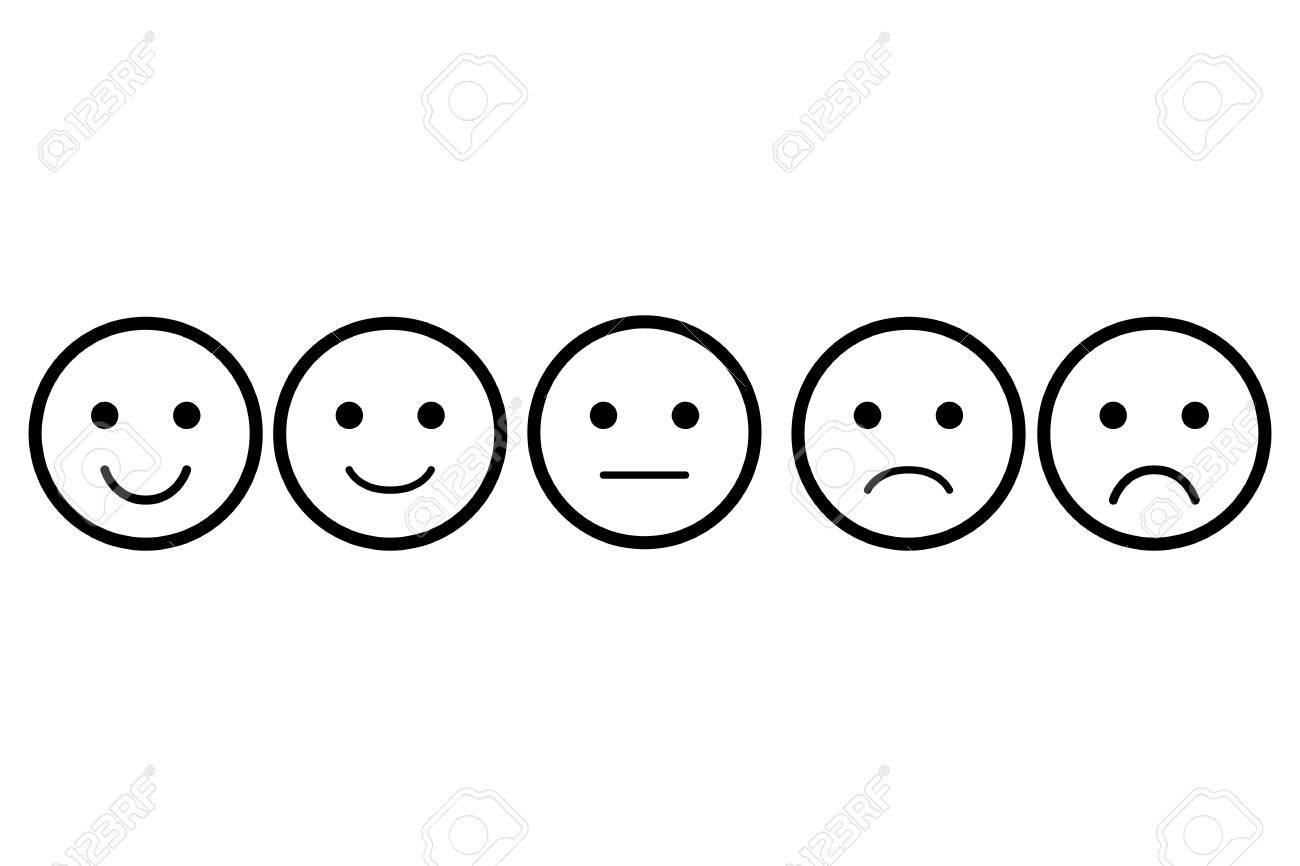


**New Song Title**: _____________________________________________________

*"Participant likes the New Song":*

(check what corresponds according to what was observed in the participant)

Strongly disagree: ____ Disagree: ____ Neutral: ____ Agree: ____ Strongly agree: ____

**Closing: Participant favourite song title**: ______________________________________________

**Final mood** (check what applies)


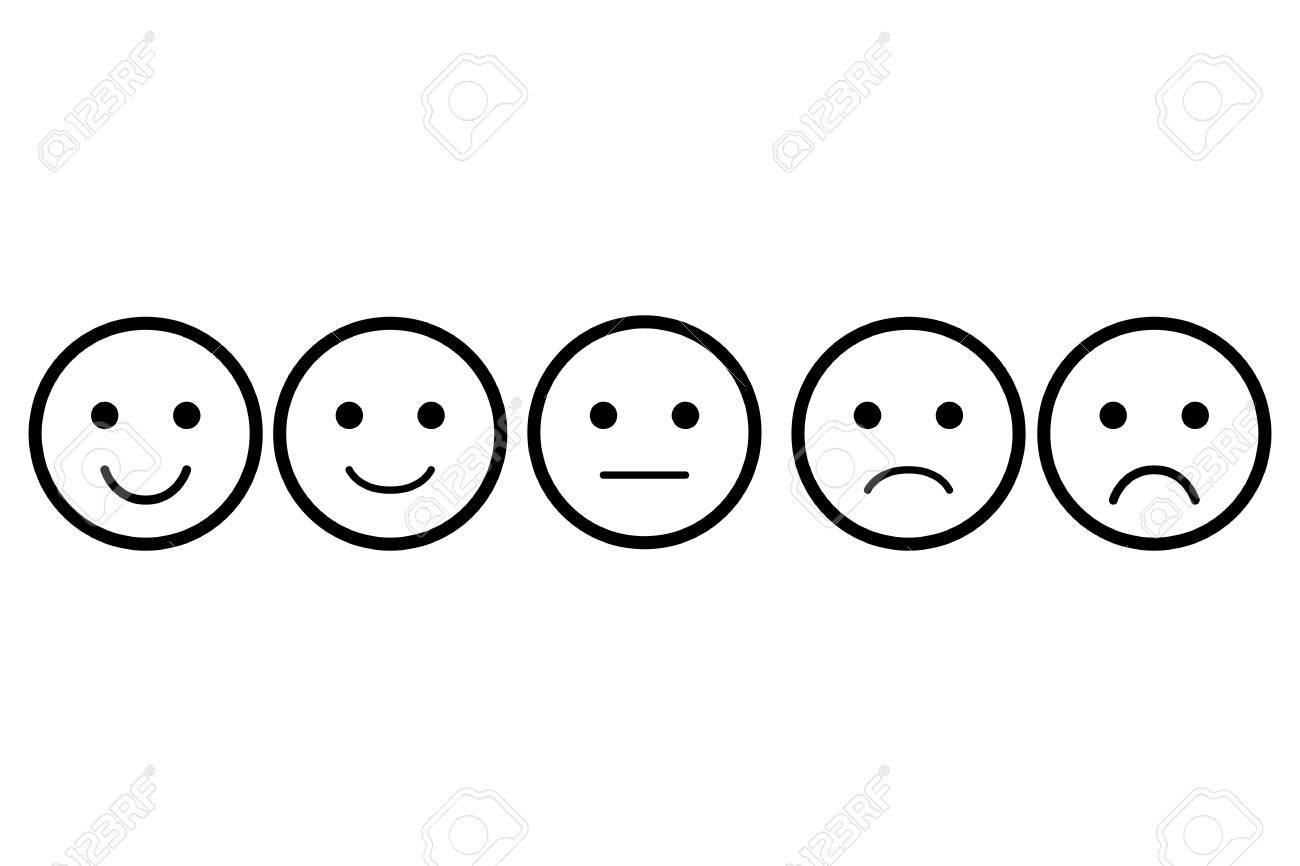


**Mark with an X where appropriate**

| Repetition | full support to sing the chorus | Intermittent support to complete each line | Cue offered:  one syllable/word per line | Only one cue/ totally independent |
| --- | --- | --- | --- | --- |
| Chorus 1 |  |  |  |  |
| Chorus 2 |  |  |  |  |
| Chorus 3 |  |  |  |  |
| Chorus 4 |  |  |  |  |
| Chorus 5 |  |  |  |  |
| Chorus 6 |  |  |  |  |
| Chorus 7 |  |  |  |  |
| Chorus 8 |  |  |  |  |
| No change chorus |  |  |  |  |
| Full Song 1 |  |  |  |  |
| Full song 2 |  |  |  |  |
| No change full song |  |  |  |  |
| Estimated Sense of Familiarity:  1 – None  2 – Implicit memory, mere exposure effect  3 – Weak familiarity  4 – Familiarity  5 – Weak recollection  6 – Recollection | | | | |

Additional comments: _____________________________________________________________

*Note.* Sense of Familiarity should be scored according to Coppalle et al. (2020), https://doi.org/10.3233/JAD-191318

**Supplementary Form F2. Intervention Fidelity Form**

**Participant # ________________ Instructor: ____________**

**Session Date:** dd/mm/yyyy **Duration: ______** minutes **Rating Date:** dd/mm/yyyy

*1. The instructor has offered the intervention according to the guidelines described in the procedures section: Y/N*

[warm up – teaches full song or chorus first – teaches one line at the time – offers 8 repetitions of entire chorus – offers 2 repetitions of entire song – prompts participant to sing chorus independently during repetitions - closing with participant’s favourite song]

Additional comments: ________________________________________________

*2. The instructor performed each step within the time frame established in the procedure section: Y/N*

Additional comments: ________________________________________________

*3. The instructor has used the materials as described in the procedures section: Y/N*

[piano/keyboard/guitar/accordion – lyrics of the chorus printed in font Arial 20 and Caps letters – full song printed in font Arial 20 Caps letters]

Additional comments: ________________________________________________

*4. The instructor establishes an empathic relationship with the participant, provides appropriate support to engage the participant and waits until the participant is ready to perform the next step of the training: Y/N*

Additional comments: ________________________________________________

**Supplementary Form F3. Participant Performance Cue Indicator Form (PPCIF)
Used by external evaluator for scoring sessions**

**Participant # ________________ Instructor: ____________**

**Session Date:** dd/mm/yyyy **Rating Date:** dd/mm/yyyy **Duration: _______** minutes

| **Number of repetitions of the chorus** | **Full support to sing the chorus**  **score: 1** | **Intermittent support to complete each line**  **score: 2** | **Cue offered: maximum one syllable/word per line**  **score: 3** | **Totally independent / only one cue**  **score: 4** | **exit**  **(score per row)** |
| --- | --- | --- | --- | --- | --- |
| **1** | **X** | **total number of times** | **line n:  word number  (word number for each cued line of chorus)** | **X** |  |
| **2** |  |  |  |  |  |
| **3** |  |  |  |  |  |
| **4** |  |  |  |  |  |
| **5** |  |  |  |  |  |
| **6** |  |  |  |  |  |
| **7** |  |  |  |  |  |
| **8** |  |  |  |  |  |
| **SCORE Chorus solo:** | | | | | **min 8 –**  **max 32** |
| **Full song 1** | **X** | **total number of times** | **line n: word number**  **(word number for each cued line of chorus)** | **X** |  |
| **Full song 2** |  |  |  |  |  |
| **SCORE Chorus in context:** | | | | | **min 2 –**  **max 8** |
| Estimated Sense of Familiarity:  1 – None  2 – Implicit memory, mere exposure effect  3 – Weak familiarity  4 – Familiarity  5 – Weak recollection  6 – Recollection | | | | | |

*Note.* Sense of Familiarity should be scored according to Coppalle et al. (2020), https://doi.org/10.3233/JAD-191318
