# Supplementary material for: Memory for Music (M4M) protocol for an international randomised controlled trial: effects of individual intensive musical training based on singing in non-musicians with Alzheimer’s disease
Source: BMJ Open. 2025 Oct 20;15(10):e095136. doi: 10.1136/bmjopen-2024-095136 (PMC12542587; doi:10.1136/bmjopen-2024-095136)
Supplement: online supplemental file 2 [file bmjopen-15-10-s002.pdf]

NORCE Norwegian Research Centre AS

versjon 4

VIL DU DELTA I FORSKNINGSPROSJEKTET HUKOMMELSE FOR MUSIKK

FORMÅLET MED PROSJEKTET OG HVORFOR DU BLIR SPURT

Dette er et spørsmål til deg om å delta i en studie om hukommelse og musikk. Ønsker du muntlig informasjon eller tilleggsinformasjon, kan du kontakte studiekoordinator, Elias Langeland (elil@norceresearch.no).

Du har blitt identifisert som mulig deltaker til denne studien. Vi kontakter mulige deltakere gjennom sykehus, dagsentre og fastleger. Helsepersonell på disse institusjonene bidrar til rekruttering av deltakere og gir informasjon om studien til mulige deltakere. Mulige deltakere som hører om studien gjennom media kan også ta kontakt mer forskerteamet direkte. Alle mulige deltakere blir satt i kontakt med noen fra forskningsgruppen for å få mer informasjon om studien og for å forsikre oss om at de oppfyller kravene for å delta i studien.

Hva er formålet med studien?

Vi ønsker å undersøke om mennesker med Alzheimers sykdom kan oppnå bedret funksjon gjennom innlæring av nye sanger sammen med en musikkterapeut. Vi bruker elektroencefalografi (EEG) og andre målemetoder for å undersøke om musikklæring virker positivt på hukommelse for sanger, kognisjon, humør, og annet som er viktig i hverdagen. EEG er en metode for å måle din hjerneaktivitet ved hjelp av at du har en hette med sensorer på hodet.

Hvem har ansvar for studien?

Studien gjennomføres av NORCE (Norwegian Research Centre AS – Bergen) i samarbeid med Universitetet i Bergen; andre samarbeidspartnere i Norge (Kinn kommune, Oslo kommune, Norsk forening for musikkterapi, Nasjonalforeningen Oslo Demensforening); og universiteter i utlandet (University of Business and Social Sciences, Buenos Aires, Argentina; Universitetet i Wien, Østerrike). Ansvarlig studieleder i Bergen er Prof. Christian Gold, NORCE. Studien gjennomføres parallelt i Norge, Argentina og Østerrike, og tar sikte på å inkludere totalt 113 deltakere, hvorav 40 vil være fra Norge.

Hva innebærer PROSJEKTET for deg?

Forskningsprosjektet vil vare totalt 12 måneder for deg som deltaker. Ved at du deltar i forskningsprosjektet, vil du være med på å gi et viktig grunnlag for videre forskning og hvordan man videre kan bruke musikk og sang i hverdagen til andre i samme situasjon som deg.

Du vil ha en egen musikkterapeut med kunnskap om Alzheimers sykdom, som kommer hjem til deg eller et annet sted som passer for deg. Gjennom sangen og

undersøkelsene vil vi finne ut om sang og musikk har en positiv virkning på ditt humør, din tankeevne, din livskvalitet, og din funksjon i hverdagen.

Du vil få tilbud om å lære sanger av en musikkterapeut over to perioder. Hver periode varer i 5 måneder. Hvis du ønsker å delta vil du få tilbud om sangtimer i begge periodene, en av dem med sangtimer to ganger per uke og den andre perioden med en sangtime per måned.

Det vil være to måneders pause mellom de to periodene.

Vi vil også gjennomføre kliniske undersøkelser, og undersøke din hjerneaktivitet med EEG.

Undersøkelsene gjennomføres på følgende tidspunkter:

- I forkant av første periode med sangtimer
- Etter 5 måneder, når første periode med sangtimer er ferdig
- Etter 7 måneder, før den andre perioden med sangtimer starter
- Etter 12 måneder, etter at andre periode med sangtimer er ferdig

I studien vil vi sammenligne effekten av perioden med sangtimer to ganger per uke, med perioden med sangtimer en gang i måneden.

Det vil tas videopptak av sangtimene. Disse videoopptakene brukes til å sørge for at musikktreningen gjennomføres slik den er tenkt, og til å vurdere dine emosjoner og din trivsel. Opptakene blir bare tilgjengelige for forskere som er involvert i studien.

Undersøkelser

Kliniske undersøkelser

Undersøkelsene vil omfatte følgende vurderingskalaer og spørreskjema:

- En undersøkelse (ADAS-Cog) vil vurdere din kognitive funksjon ved Alzheimers sykdom: Du vil her få oppgaver og spørsmål for å undersøke hvordan du oppfatter, husker og benytter deg av ulik type informasjon. Dette vil vurderes fire ganger i løpet av prosjektet – ved oppstart og etter 5, 7, og 12 måneder. Denne delen av undersøkelsen vil vare mellom 30 og 45 minutter.
- På første møte vil du også få oppgaver og spørsmål fra en kortere (10 minutter) test (Mini-Mental State Examination). Dette er en test som leger ofte benytter når de møter en pasient med Alzheimers sykdom. Du vil dessuten få spørsmål om din diagnose, alder, kjønn, nasjonalitet, familiestatus, om du bor i en vanlig eller tilpasset leilighet, og om din erfaring med musikk.

EEG

- Måling av hjerneaktivitet: Du vil få en hette med sensorer på hodet ditt. Sensorene vil bli fylt med en gel for å skape en stabil forbindelse mellom sensorene og huden din. Vi vi be deg om å lytte på musikkstykker mens du har hetten på. Undersøkelsen vil gjennomføres fire ganger – ved oppstart og etter

5, 7, og 12 måneder i prosjektet. Denne delen av undersøkelsen vil vare omtrent en time (inkludert forberedelse av EEG-hetten og EEG-måling).

MULIGE FORDELER OG ULEMPER

Ved å delta i studien vil du bidra til å gi et viktig grunnlag for videre forskning og videreutvikling av metoder for musikalsk trening for eldre personer. Etter studiens slutt vil vi gi informasjon om resultater og publikasjoner fra prosjektet.

Det er ingen kjent risiko eller kjente bivirkninger av de metodene som benyttes i studien.

EEG-undersøker har vært i bruk i mange år, og vi kjenner ikke til risiko eller bivirkninger knyttet til å delta i slike undersøkelser. Det er likevel viktig at du sier ifra til oss om det er noe du lurer på i forbindelse med undersøkelsen.

Under undersøkelsen vil du sitte godt i en stol mens en spesiell hette med små sensorer fanger opp aktivitet i hjernen din. Etter undersøkelsen tas hetten av. Om du ønsker det, kan vi vaske håret ditt.

Vi gjør ingen opptak som kan brukes til klinisk diagnostikk. EEG-undersøkelsen som gjennomføres i prosjektet kan derfor ikke avdekke sykdom.

FRIVILLIG DELTAKELSE OG MULIGHET FOR Å TREKKE DITT SAMTYKKE

Det er frivillig å delta i prosjektet. Dersom du ønsker å delta, undertegner du samtykkeerklæringen på siste side. Du kan når som helst og uten å oppgi noen grunn trekke ditt samtykke. Det vil ikke ha noen negative konsekvenser for deg hvis du ikke vil delta eller senere velger å trekke deg.

Du kan IKKE delta i undersøkelsen hvis du

- er yngre enn 65 år;
- ikke har dokumentasjon fra lege om symptomer forenlig med Alzheimers sykdom;
- bor på sykehjem;
- har eller har hatt arbeid som musiker.

Så lenge du kan identifiseres i datamaterialet, har du rett til å få se opplysningene vi har om deg, og rette eller slette opplysninger som er feil eller misvisende. Du kan kreve å få en kopi av opplysningene innen 30 dager, og du kan klage på behandlingen av dine personopplysninger til Datatilsynet eller NORCE sitt personvernombud. Du har også rett til å få innsyn i sikkerhetstiltakene ved behandling av opplysningene. Å kreve adgang, sletting, eller utlevering gjelder ikke dersom materialet eller opplysningene er anonymisert eller publisert. Denne adgangen kan også begrenses dersom dataene er bearbeidet eller brukt i analyser.

Dersom du senere ønsker å trekke deg eller har spørsmål til prosjektet, kan du kontakte prosjektkoordinator eller prosjektleder (se kontaktinformasjon på siste side).

HVA SKJER MED OPPLYSNINGENE OM DEG?

Opplysningene som registreres om deg skal kun brukes som beskrevet i dette dokumentet, og planlegges brukt fram til 2030. Eventuelle utvidelser i bruk og oppbevaringstid kan kun skje etter godkjenning fra Regional etisk komité (REK) og andre relevante myndigheter.

Alle opplysningene vil bli behandlet uten navn og fødselsnummer eller andre direkte gjenkjennende opplysninger. En kode knytter deg til dine opplysninger gjennom en navneliste, og kun autorisert personell knyttet til prosjektet har tilgang til denne listen. Dermed forblir det personlige data og er derfor underlagt databeskyttelse som er regulert gjennom GDPR-regelverket.

Resultatene av studien skal kun brukes i forskningssammenheng og ingen av resultatene som senere vil publiseres kan spores tilbake til deg.

Opplysningene om deg vil bli oppbevart på et sikret sted i fem år etter prosjektslutt av kontrollhensyn. Disse opplysningene vil bli slettet i sin helhet etter den perioden.

DELING AV OPPLYSNINGER OG OVERFØRING TIL UTLANDET

Ved å delta i prosjektet, samtykker du også til at kodede opplysninger om deg som alder, kjønn, musikalsk treningsforløp (inkludert videoopptak fra musikalske treningsøkter), og EEG-undersøkelsen kan overføres til utlandet som ledd i forskningssamarbeid og publisering. Koden som knytter deg til dine personidentifiserbare opplysninger vil ikke bli utlevert.

Etter prosjektslutt vil avidentifiserte kliniske data og EEG-data bli gjort tilgjengelig for gjenbruk av andre forskere via en internasjonal database (Open Science Foundation, OSF.io) og i forskernettverk, for å muliggjøre forskning som fremmer forståelse av kognitive vansker ved Alzheimers sykdom.

FORSIKRING

NORCE har særskilt bedrifts- og produktansvarsforsikring; formueansvarsforsikring; og yrkesskadeforsikring.

GODKJENNINGER

Regional komité for medisinsk og helsefaglig forskningsetikk har gjort en forskningsetisk vurdering og godkjent prosjektet. [no. xxxx].

NORCE og prosjektleder Prof. Christian Gold (NORCE) er ansvarlig for personvernet i prosjektet.

Vi behandler opplysningene konfidensielt basert på NORCE sine standardrutiner for sikker databehandling og i samsvar med personvernregelverket (bl.a. EUs personvernforordning, GDPR). Kontaktopplysningene og svarene på spørreskjemaene vil lagres på en sikker server ved Universitetet i Bergen (UiB SAFE) frem til de vil bli anonymisert (kontaktopplysninger slettes). Norsk senter for forskningsdata AS (SIKT) har vurdert at behandlingen av personopplysninger i dette prosjektet er i samsvar med personvernlovgivningen.

KONTAKTOPPLYSNINGER

Dersom du har spørsmål til prosjektet eller ønsker å trekke deg fra deltakelse, kan du kontakte:

Prosjektkoordinator Elias Langeland, telefon 906 28 887, epost elil@norceresearch.no

Prosjektleder Christian Gold, telefon 975 01 757, epost chgo@norceresearch.no

Dersom du har spørsmål om personvernet i prosjektet, kan du kontakte personvernombudet ved institusjonen: personvernombud@norceresearch.no

SAMTYKKE TIL Å BLI KONTAKTET OM OPPFØLGINGSPROSJEKT

I tillegg ønsker vi å kontakte deg i fremtiden, dersom det blir aktuelt med et oppfølgingsprosjekt. Dette er ikke en forutsetning for å delta i denne studien. Kryss av under dersom du ønsker å samtykke til dette.

☐ Jeg samtykker til at forskere knyttet til dette prosjektet kan kontakte meg i fremtiden om eventuelle oppfølgingsprosjekt.

SAMTYKKE TIL Å DELTA I STUDIEN

Jeg samtykker til å delta i prosjektet og til at mine personopplysninger brukes slik det er beskrevet.

Sted og dato Deltakerens signatur

Deltakerens navn med trykte bokstaver

Jeg bekrefter å ha gitt informasjon om prosjektet

Sted og dato Signatur

Fagpersonens navn og rolle i prosjektet
