# Supplementary material for: Memory for Music (M4M) protocol for an international randomised controlled trial: effects of individual intensive musical training based on singing in non-musicians with Alzheimer’s disease
Source: BMJ Open. 2025 Oct 20;15(10):e095136. doi: 10.1136/bmjopen-2024-095136 (PMC12542587; doi:10.1136/bmjopen-2024-095136)
Supplement: Supplementary file 3 [file bmjopen-2024-095136.reviewer_comments.pdf]

PEER REVIEW HISTORY

BMJ Open publishes all reviews undertaken for accepted manuscripts. Reviewers are asked to complete a checklist review form and are provided with free text boxes to elaborate on their assessment. These free text comments are reproduced below.

ARTICLE DETAILS

Title (Provisional)

Memory for Music (M4M) protocol for an international randomized control trial: Effects of individual intensive musical training based on singing in non-musicians with Alzheimer´s Disease

Authors

Lichtensztejn, Marcela; Cui, Anja-Xiaoxing; Geretsegger, Monika; Lundervold, Astri; Koelsch, Stefan; Pfabigan, Daniela; Assmus, Jörg; Langeland, Elias; Ruiz, Marianna; Tabernig, Carolina; Skogseth, Ragnhild Eide; Gold, Christian

---

VERSION 1 - REVIEW

Reviewer 1

Name Hoare, Zoe

Affiliation Bangor University, North Wales Organisation for Randomised Trials in Health

Date 29-Nov-2024

COI None

Understand the statistical premise of using a cross-over design for an evaluation but although a wash-out period is included I'm not convinced learning effects would not have laid over thus not truly testing two independent treatments within the design but the ordering of treatments within an individual.

I'm not convinced the sample size has been described adequately particularly justification of the correlation between interventions, which if considered within an parallel group ANCOVA calculation would provide the same sample size. This coupled with the learning effects led me to query the cross-over design as an evaluation of the two interventions and rather a comparison of the order of two interventions. Further justificaiton and clarification on the design would be useful for the reader due to this being a less standard design.

Reviewer 2

**Name**	**Yuan, Shauna H.**
**Affiliation**	**University of Minnesota**
**Date**	**30-Mar-2025**
**COI**	**None**

The authors aim to fill the gap about the effects of active music interventions on cognition in research by measuring the effects of learning new songs on cognitive functioning. M4M will examine memory for new songs in non-musician adults with AD after undergoing intensive versus minimal individual musical training on singing novel songs. This is an international study in three different countries Norway, Argentina and Austria. The manuscript is clearly written.

Some clarifications are recommended:

1. Inclusion criteria for ADAS-Cog, is there a cut-off or range?

2. Is there any plan in balancing the two sexes?

3. How are the song chosen? Is there a list of songs?

4. How do the authors account for missing data?

**Reviewer**	**3**
**Name**	**Petrovsky, Darina V.**
**Affiliation**	**Duke University School of Nursing**
**Date**	**07-Apr-2025**
**COI**	**None**

Thank you for the opportunity to review this manuscript. The strengths of the manuscript are: 1) well-described intervention protocol; 2) adequate description of main outcomes and procedures; and 3) solid discussion. The weaknesses are: 1) introduction does not adequately address the need to examine cognitive outcomes in music interventions; and 2) why it is important to examine mediators (vs moderators such as mood) of the response and 3) lack of clarity of why the proposed intervention is innovative.

VERSION 1 - AUTHOR RESPONSE

Reviewer: 1
Dr. Zoe Hoare, Bangor University
Comments to the Author:

R1Q1: Understand the statistical premise of using a cross-over design for an evaluation but although a wash-out period is included I'm not convinced learning effects would not have laid over thus not truly testing two independent treatments within the design but the ordering of treatments within an individual.
RESPONSE: Thank you for your review and pointing out aspects of our study descriptions that were not clear enough. We have clarified in the revised manuscript that although the interventions occur sequentially, we mitigate concerns about carry-over effects in several ways. First, we include a two-month wash-out period between phases. Second, we use different musical pieces in each phase, so participants are not continuing work on the same material. Third, the statistical model can include the order of interventions as a covariate to detect and account for possible sequencing effects. The goal of the trial is not to test the order of treatments, but rather to compare the effects of different *intensities* of the same musical training approach on learning and cognitive outcomes. See the first paragraph of the 'Trial design and procedures' section.
R1Q2: I'm not convinced the sample size has been described adequately particularly justification of the correlation between interventions, which if considered within an parallel group ANCOVA calculation would provide the same sample size.
RESPONSE: Thank you for this helpful comment. We have clarified in the manuscript that the assumed correlation (r = 0.5) represents a conservative estimate of within-subject consistency across intervention phases, based on typical expectations in repeated cognitive assessments for similar populations. While baseline adjustment in a parallel-group ANCOVA also improves power, the crossover design offers additional benefits through within-subject comparison, which more effectively reduces variance and thus reduces required sample size. This distinction has been added to the 'Sample size and power' section.
R1Q3: This coupled with the learning effects led me to query the cross-over design as an evaluation of the two interventions and rather a comparison of the order of two interventions. Further justification and clarification on the design would be useful for the reader due to this being a less standard design.
RESPONSE: Thank you for this thoughtful observation. We have revised the 'Trial design and procedures' section to further clarify that our study does not compare two fundamentally different interventions, but rather two levels of intensity (minimal vs. intensive) of the same musical training approach. While crossover designs are less common in psychosocial interventions, they are well suited to our aim, which is to test whether increased time and engagement with musical material leads to greater cognitive and behavioral effects. As noted in response to R1Q1, we have addressed concerns about carry-over by using different musical pieces in each phase, implementing a wash-out period, and including the order of interventions as a covariate in our statistical model. We hope this revised explanation helps clarify the rationale behind the design for readers.

Reviewer: 2
Dr. Shauna H. Yuan, University of Minnesota
Comments to the Author:

The authors aim to fill the gap about the effects of active music interventions on cognition in research by measuring the effects of learning new songs on cognitive functioning. M4M will examine memory for new songs in non-musician adults with AD after undergoing intensive versus minimal individual musical training on singing novel songs. This is an international study in three different countries Norway, Argentina and Austria. The manuscript is clearly written.<br>Some clarifications are recommended:
RESPONSE: Thank you for your supportive assessment and helpful comments. We have responded to each of the issues raised below.

R2Q1: 1. Inclusion criteria for ADAS-Cog, is there a cut-off or range?
RESPONSE: There is no cut-off for participants in M4M; all those who fulfil inclusion criteria are assessed according to standard procedure. This is stated now explicitly in 'Eligibility criteria and enrolment'. We had initially a cutoff for MMSE (MMSE < 24 was mentioned in the grant proposal) but dropped that in favour of clinical criteria. This is mentioned in Table 1, which is cited in the same section.
R2Q2: 2. Is there any plan in balancing the two sexes?
RESPONSE: Based on experiences from similar previous studies, we anticipate a higher proportion of female participants, which probably reflects different prevalence rates in the population. The primary goal of this study is to examine intervention effects in a sample that is generalisable to the population. Balancing sexes would maximise power for comparing sexes but would weaken the generalisability of the sample. Therefore, we believe that balancing sexes is not the best strategy to serve the goals of this study. We have not changed the manuscript in response to this comment but would be willing to add a statement that a higher number of females may be expected.
R2Q3: 3. How are the song chosen? Is there a list of songs?
RESPONSE: We have now rephrased the first para of 'Interventions' to make clear that songs are not chosen for the study from a list of existing songs but newly composed for the study based on certain criteria regarding musical complexity; and that they are randomly chosen from that list of novel songs. That the order in which those randomly chosen songs will be learned is also randomised was already stated in the second para of 'Interventions'.
R2Q4: 4. How do the authors account for missing data?
RESPONSE: The statistical model specified in 'Statistical analysis' requires complete data for all 4 time points. Multiple imputations of outcomes is normally not recommended because the model we specified (ANCOVA with participant and site as random effects, i.e. a type of LME) already makes optimal use of all available data. We have added this, and have also specified that a detailed SAP will be developed before the start of analyses.

Reviewer: 3
Dr. Darina V. Petrovsky, Duke University School of Nursing
Comments to the Author:

Thank you for the opportunity to review this manuscript. The strengths of the manuscript are: 1) well-described intervention protocol; 2) adequate description of main outcomes and procedures; and 3) solid discussion.
RESPONSE: Thank you for this positive appraisal of our manuscript.
The weaknesses are: 1) introduction does not adequately address the need to examine cognitive outcomes in music interventions;
RESPONSE: We have now added more arguments and references in relation to music, music-making and cognition to further support the need of studying the effects of music training in memory and general cognition in individuals with AD, and made sure that the introductory paragraph highlights cognition as a central feature of AD. (See 'Introduction', first 3 paragraphs.)
and 2) why it is important to examine mediators (vs moderators such as mood) of the response
RESPONSE: As stated in the revision of our manuscript (first pararaph of 'Introduction'), progressive cognitive deterioration in AD is closely interrelated to changes in emotional domains.
and 3) lack of clarity of why the proposed intervention is innovative.
RESPONSE: The most important innovative aspect is the use of novel songs. Most if not all previous studies have relied on reminiscence, i.e. using biographically relevant music that the participant remembers from their youth. Given that previous brain research has suggested a distinct area for music memory, and a recent case study on which this study builds has suggested an ability of some AD patients to learn novel songs, it will be very relevant to test this ability in a rigorous study. If successful, it may yield new possibilities for preventing cognitive decline. This is now clarified in several sections: 'Strengths and limitations'; 'Introduction', paragraphs 3 and 5.
